# Case Illustrating the Nature of Epilepsy

**Published:** 1868-10

**Authors:** L. Marowsky

**Affiliations:** Chareow, Russia


### CASE ILLUSTRATING THE NATURE OF EPILEPSY.

By Prof. L. MAROWSKY, of Chareow, Russia.

This case is reported as a further contribution to the researches of Dr. Nothnagel (cf. p. 67 of this volume) concerning the origin of the epileptic attack, and the author is of opinion that it proves beyond controversy that many, if not all, forms of epilepsy are owing to a spastic contraction of the cerebral arteries; in other words, that epilepsy is a *vasomotor neurosis of the brain.* The arterial spasm may be induced by various causes, either directly or by reflex action; hence the multifarious forms and modifications of the disease. Whenever these causes are known, or discovered *post mortem*, we speak of "symptomatic epilepsy;"—when they remain unknown, we call the disease "essential" (idiopathic) epilepsy.

The case is that of a cadet, æt. 16, with a moderate phlegmenous inflammation extending from the left ala of the nose to the middle of the left cheek. The young man was naturally very excitable, nervous, but of strong build and in good condition. The tumor was very painful, at the circumference hard and red; the centre was dark-red, almost livid, and showed fluctuation so distinct as to indicate the propriety of opening the abscess. This little operation was performed immediately, the patient standing. A small, but sufficient, opening had scarcely been made with a sharp lancet, when the skin immediately around the incision, which had been dark-red, became perfectly white. This white margin rapidly extended farther and farther, all of the red inflamed surface became as white as chalk, the patient became restless, his pupils dilated and his countenance grew pale; he fell, lost consciousness, and, after a slight tetanic contraction, was attacked with clonic convulsions of the face, hands, and feet, and foaming at the mouth. After a half or at most one minute, the convulsions ceased, the patient became quiet, his face reddened, the pulse, which had been slow, contracted and tense, became frequent and easily compressible, the skin moist, and after five or ten minutes the patient awoke, remembered nothing of the accident, and for the rest of the day remained depressed, feeble, and incapable of mental exertion.

The author supposes that he had caused, by the incision, a reflex spasm of the cerebral arteries, leading to general anæmia of the organ, which then caused convulsions and loss of consciousness. The tonic contraction of the cerebral vessels must have been general, affecting the base as well as the periphery, because loss of consciousness occurred simultaneously with general tonic and clonic convulsions.

The patient has been under observation for two years since this occurrence, but never had a second attack; nor had he ever suffered from an epileptic attack previously, though he had always been very irritable as a boy.—*Deutsches Archiv f. klin Med.*, vol. iii., p. 615. 1867.

